# Development of visual attention control in early childhood: Associations with temperament and home environment

**DOI:** 10.3389/fpsyg.2022.1069478

**Published:** 2022-12-21

**Authors:** Sebastián Moyano, Ángela Conejero, María Fernández, Francisca Serrano, M. Rosario Rueda

**Affiliations:** ^1^Department of Experimental Psychology, University of Granada, Granada, Spain; ^2^Mind, Brain and Behavior Research Center (CIMCYC), University of Granada, Granada, Spain; ^3^Department of Developmental and Educational Psychology, University of Granada, Granada, Spain

**Keywords:** endogenous attention, monitoring, temperament, executive attention, toddlerhood, preschool, home chaos

## Abstract

Endogenous visual attention orienting is early available from infancy. It shows a steady development during the preschool period towards monitoring and managing executive attention to optimize the interplay between environmental contingencies and internal goals. The current study aims at understanding this transition from basic forms of endogenous control of visual orienting towards the engagement of executive attention, as well as their association with individual differences in temperament and home environment. A total of 150 children between 2 and 4 years of age were evaluated in a Visual Sequence Learning task, measuring visual anticipations in easy (context-free) and complex (context-dependent) stimuli transitions. Results showed age to be a predictor of a reduction in exogenous attention, as well as increased abilities to attempt to anticipate and to correctly anticipate in complex transitions. Home chaos predicted more complex correct anticipations, suggesting that the exposure to more unpredictable environments could benefit learning in context-dependent settings. Finally, temperamental surgency was found to be positively related to sustained attention in the task. Results are informative of age differences in visual attention control during toddlerhood and early childhood, and their association with temperament and home environment.

## Introduction

Attention is key for selecting the relevant information from the environment and controlling both information processing and behavior (Posner and Rothbart, 1998). Classical (Posner, 1980; Posner and Petersen, 1990) as well as more recent (Corbetta and Shulman, 2002) models of attention differentiate between exogenous and endogenous control of attention. Exogenous orienting is a bottom-up process that occurs when salient stimuli or changes in the environment draw and direct attention automatically (i.e., stimulus-driven). This type of orienting differs from shifts of attention that are based on expectancies or internal goals, which are referred to as endogenous or top-down orienting. The central role of attention within the cognitive system makes the development of this function crucial to children’s learning. Further, attention is related to other spheres of the child’s functioning during development, such as academic achievement and socio-emotional adjustment (Simonds et al., 2007; Rueda et al., 2010). These associations are found from early infancy (Rothbart et al., 2011; Blankenship et al., 2019), and appear to be predictors of children’s functioning in adulthood (Moffitt et al., 2011).

Previous research on the early development of visual attention has mainly focused on initial transitions from exogenous to endogenous forms of control in infancy. Evidence suggests that primary aspects of visual exogenous orienting emerge early in life. At around 1 month of age, infants are already able to fixate on and follow moving stimuli in the absence of visual competition (Aslin, 1981; Atkinson and Braddick, 1985), with changes in exogenous control as top-down processes gains weight over volitional control. However, changes with age in exogenous attention are still unclear in early childhood. In this sense, Ristic and Kingstone (2009) found that exogenous orienting of 3-to 6-year-olds children upon non-predictive cues was similar to adults. Nevertheless, Iarocci et al. (2009) reported that 5-year-olds children displayed a higher tendency to engage exogenous orienting in comparison to 7, 9, and 24-year-olds participants. This suggests that as older cohorts gain control over endogenous orienting, it will allow for a modulation of exogenous attention. Yet, no previous research has covered changes in exogenous orienting between toddlerhood and preschool ages, in relation to changes in endogenous control.

Conversely, the development of endogenous orienting is more protracted in time, emerging around 3-to 6 months of age (Johnson et al., 1991; McConnell and Bryson, 2005). The recruitment of crucial areas for endogenous attention control, such as frontal and parietal regions, have been found already in 3-month-olds infants (Ellis et al., 2021). Although, endogenous control seems to show an increased stability from the preschool years onwards (Rothbart et al., 2003; Colombo and Cheatham, 2006). The relevance of endogenous orienting also lies in its conception as a precursor for the development of more complex mechanisms for attention control, such as those involved in the voluntary regulation of thoughts and behavior, particularly in interference-rich contexts. These mechanisms are known to be mostly dependent on executive attention (Rothbart et al., 2011), which shares common neural substrates with endogenous orienting (Rueda et al., 2015). One of these more sophisticated mechanisms of control that has been scarcely studied during toddlerhood and preschool is referred to as context monitoring. Monitoring describes the ability to track the course of events (Petersen and Posner, 2012), and is related to the quality and flexibility with which attention control is engaged, providing a more effective detection of these target events (Chevalier and Blaye, 2016). Moreover, it is a crucial control process during learning and memory creation (Nelson and Narens, 1990), as tracking the events that occur around us is a necessary condition to form expectations about the environment. This is particularly important in more complex settings, allowing to orient the attentional focus toward areas or objects of interest in rich but predictable environments.

Previous research has found that since toddlerhood, children are already able to monitor the environment in search of regularities to create expectations. To study this, Rothbart et al. (2003) presented visual sequences of spatial locations to toddlers from 2-to 3 years of age. They found that 2-year-olds were already able to monitor complex sequences, in which the location of the next stimulus was correctly predicted only if the child was able to monitor the previous location to the current one, that is engaging mechanisms of context monitoring. However, between 3 and 5 years of age, Freier et al. (2017) showed that the ability of children for sequence monitoring is still evolving. In their study, children’s goal-oriented behavior was measured employing a sequence coloring task, with children being required to use a set of colors equally often while coloring all the animals in a sequence presented on paper. They found younger children to deviate earlier in the task from goal-directed responses compared to 5-year-olds, suggesting a lower ability to monitor the distal goal of the task. Also, it is likely related to age differences in the monitoring of previous actions, that is colors already used, for the implementation of future steps. Differences in task performance were not found to be related to working memory capacity, but to the engagement of endogenous attention control during the selection of the appropriate actions at lower-levels of task requirements to achieve the final goal.

Due to the relevance of attention during development, in the current research we aimed to study the transition from exogenous to endogenous orienting to context monitoring through the visual modality. For this, we evaluated children from 2-to 4 years of age, a developmental period in which executive attention is proposed to start emerging as the main supervisory system of attention (Posner and Rothbart, 2007; Rothbart et al., 2011). Is this transition towards executive attention which supports the engagement of more sophisticated mechanisms of control. We aim at doing this with a single task: the Visual Sequence Learning (VSL).

### Measuring attention orienting during development

The so-called visual expectation paradigm (VExP; Haith et al., 1988) is one of the first experimental protocols suitable for infants and young children that allows for the measurement of both exogenous and endogenous orienting of attention. This paradigm involves the presentation of a set of visual stimuli in different spatial locations, following a fixed sequence, while the direction of the participants’ gaze is being recorded. Exogenous shifts of attention are measured through reactive looks, which are observed after the stimulus onset. In contrast, anticipatory looks to a particular location, that is before the stimulus onset, reflect an expectancy-based endogenous orienting of attention. The accuracy of anticipatory looks hinges on whether there is an effective learning of the regularities available in the context, such as the repeated sequence of events (Rothbart et al., 2003).

To detect these regularities, sustained attention and context monitoring are key abilities that will drive the detection and knowledge acquisition of events’ frequencies, in order to form accurate expectations of upcoming occurrences. To explore higher-level attention mechanisms involved in monitoring during sequence learning, Clohessy et al. (2001) developed the VSL. The procedure of the VSL involves the presentation of a number of attractive events in different spatial locations that appear in a fixed sequence. A common one used with toddlers is 1-2-1-3-1-2-1-3-and so on, where each number represents a particular location. The original study employed a set of three screens to define each spatial location, placing a camera between them to record infants’ gaze. Similar to the VExP, it enables to measure exogenous orienting through reactive looks. Furthermore, this particular sequence allows for the distinction of anticipatory looks during easy (unambiguous) transitions, given that locations 2 and 3 are always followed by location 1, and complex (ambiguous) transitions, given that location 1 can be followed by location 2 or 3, depending on the previous location to 1. Thus, complex transitions require a greater engagement of context monitoring processes (i.e., maintaining information in mind about previous locations) in order to correctly anticipate the location of the upcoming event.

The VSL was designed as a suitable task for children of different ages. As no verbal instructions are needed, the experimental protocol is free of limitations due to instructions comprehension. It has been used with infants from 4 months of age (Clohessy et al., 2001; Sheese et al., 2008), although only Rothbart et al. (2003) focused on studying age differences, specifically between 2 and 3 years of age. In a cross-sectional study, Clohessy et al. (2001) analyzed each cohort independently studying differences between easy and complex transitions for 4, 10 and 18-month-olds, as well as adults. In a single exposition to the task, they found that 4 and 18-month-olds showed a similar percentage of anticipatory looks in easy transitions to adults. However, only adults showed differences between easy and complex transitions, showing more correct anticipations to complex compared to easy. In a further study, Rothbart et al. (2003) found that it is between 2 and 3 years of age when children appear to exhibit an increase in correct anticipations for complex but not for easy transitions. This indicates that compared to complex, easy transitions require less endogenous control of attention reaching adult-like levels earlier in infancy, while increases in complex transitions are protracted until toddlerhood.

The resolution of ambiguity, such as the one that complex transitions require, demand greater attentional control. In sequence-learning studies with adults, employing a key press response instead of anticipatory gaze, participants fail to learn the sequence when they perform a concurrent task that demands attentional resources (Curran and Keele, 1993). It has been proposed that solving this ambiguity to correctly anticipate in complex transitions provides a measure of context monitoring, a supervisory process associated with a higher attentional control provided by executive attention (Posner and DiGirolamo, 1998; Botvinick et al., 2001). Previous results with the VSL also support this notion, as the percentage of correct anticipations in complex transitions has been found to correlate with a lower interference effect in a spatial conflict task in 3-year-old children (Rothbart et al., 2003). This suggests that endogenous orienting of attention in a task entailing context monitoring also taps into executive attention processes.

### Individual differences in attention control in relation to temperament and environmental factors

Emerging control over attention relates to multiple aspects of life, including social adjustment and academic performance during childhood (Rueda et al., 2010), as well as socioeconomic success or personal and emotional wellbeing in adulthood (Moffitt et al., 2011; Daly et al., 2015). An aspect inherent to the child and strongly associated with individual differences in attention control is temperament (Rueda et al., 2011). Effortful control (EC) is one dimension of temperament defined as the child’s capacity to exert voluntary control over reactive systems of approach (surgency and/or aggression) and withdrawal (negative affectivity, such as fear and/or shyness) (Posner and Rothbart, 2007). Previous research has shown empirical links between EC and attention control. Children scoring high in EC tend to exhibit a better attentional ability during toddlerhood (Gerardi-Caulton, 2000; Rothbart et al., 2003; Nakagawa and Sukigara, 2013) and late childhood (Simonds et al., 2007). Also, High-EC children are more able to self-regulate behavior (Kochanska et al., 2000), which in turn favors learning and context monitoring processes (Pintrich, 2000; Ursache et al., 2012). In contrast, temperamental reactive systems such as surgency (SUR) and negative affectivity (NA) have been mostly negatively related to attention and EC from very early life (Rothbart and Rueda, 2005; Rothbart et al., 2011). Surgency refers to individual differences in positive emotionality and approach, including impulsivity and sensation seeking (Posner and Rothbart, 2007). Early attention control during infancy has been found to show a negative association with SUR, but positive with EC, during childhood (Papageorgiou et al., 2014, 2015). Also, high-SUR toddlers tend to perform fewer anticipations in both easy and complex transitions in the VSL task, indicating a lower control over endogenous orienting (Rothbart et al., 2003). On the other hand, NA integrates children’s negative emotionality, including behavioral reactivity related to discomfort, anger/frustration and fear (Posner and Rothbart, 2007). This factor shows a consistent negative association with attention control in infants (Johnson et al., 1991; McConnell and Bryson, 2005), toddlers and even young children (Gerardi-Caulton, 2000; Rothbart et al., 2003).

Recent research has reported effects of not only temperament, but also in conjunction with environmental factors on early visual attentional abilities (Conejero and Rueda, 2018). Different aspects of the home environment are known to have an impact on the development of children’s attention. For instance, previous studies inform of an association between higher chaos at home and poorer executive functioning (Vernon-Feagans et al., 2016). Matheny et al. (1995) defined chaotic home environments as those characterized by high levels of background noise, crowded spaces, and disorganized timetables or lack of routines, which increase the levels of environmental confusion. It has been suggested that children exposed to home chaos are more likely to disconnect from their immediate context, as they grow up under over-stimulating conditions (Evans, 2006). However, only one study has explored the effects of chaotic environments over attention. Tomalski et al. (2017) reported detrimental effects of chaos over infants’ processing speed measured through visual attention. Their results highlight that the characteristics of the home environment play a significant role on infants’ attentional skills. Although chaos seems to be closely related to other environmental factors such as socioeconomic status (SES; Evans and Schamberg, 2009), chaos has been found to predict independent effects over early cognitive functioning than those of SES (Petrill et al., 2004; Hart et al., 2007).

### Aims and hypothesis

Endogenous attention continues to improve until at least late childhood (Abundis-Gutiérrez et al., 2014; Pozuelos et al., 2014). Nevertheless, research investigating age differences in exogenous, endogenous orienting and context monitoring between toddlerhood and preschool-ages is sparse. Analyzing children’s gaze in the VSL task allows to study differences in multiple components of visual attention control through these developmental stages. We aim to provide measures of exogenous (i.e., reactive looks) and endogenous orienting of attention (i.e., sustained attention and general anticipations) no previously reported with this paradigm. Furthermore, the dissociation between anticipatory looks in easy and complex transitions will be informative of differences in children’s endogenous orienting under different levels of context monitoring demands. We intend to do this adapting the VSL task to an eye-tracking protocol to gain in temporal and spatial precision. Measures of child’s temperament and family’s home chaos were also included in order to test their contribution to attention. To the best of our knowledge, no previous study has investigated the effects of chaos on children’s orienting of attention, neither its contribution with temperament at these ages.

For this purpose, a cross-sequential design of 5 cohorts was used. The within-and between-groups design of the study aims at testing both age differences as well as within-subject stability of attentional measures. We expected to find no contribution of age to reactive looks as a measure of exogenous orienting. Given that the task entails the presentation of a repeated number of sequences over a period of time, the percentage of stimuli fixations may provide a measure of sustained attention to the task. We hypothesized an increased percentage of stimuli fixations with age. Also, anticipatory looks provide a measure of the development of endogenous orienting as voluntary attempts to anticipate an upcoming event, independently of the accuracy of the expectation. Thus, we expected to observe an increase with age in total anticipations. In addition, we hypothesized an increase in correct anticipations with age, with a higher contribution of age to the monitoring of complex transitions. We reason that age-related increases in executive attention control would favor context monitoring of the visual sequence. However, the contribution of age for correct anticipations in easy transitions would be less prominent (see Table 1 for a summary of attentional processes measured in the VSL task). Although no *a priori* hypotheses were established, we will explore whether there is individual stability of all different attentional processes involved in the VSL task between sessions. Regarding the secondary aim of the study, we expected temperamental EC to positively contribute to endogenous orienting and context monitoring, whereas negative contributions for surgency and negative affectivity were anticipated. Concerning home environment, we hypothesized a negative contribution of chaos to children’s attentional abilities, particularly in task conditions with higher loads of context monitoring.

**Table 1:** Summary of the main dependent variables of the VSL task and their associated attentional process.

Dependent variable	Attentional process
Stimulus fixations	Sustained attention
Reactive looks	Exogenous orienting
Total anticipations	Endogenous orienting
Easy correct anticipations	Endogenous orienting-based learning
Complex correct anticipations	Endogenous orienting + monitoring-based learning

## Materials and methods

### Participants

Toddlers and young children (*n* = 150) between 2 and 4 years of age were recruited from kindergartens and primary schools in the city of Granada (Spain) and its metropolitan area. Some children were excluded due to preterm birth (i.e., before the 37^th^ gestational week; *n* = 1), suspected developmental disorder (*n* = 6) or data fuzziness (*n* = 8). The final sample of 135 (*n* = 69 female) children was divided into five age groups of 2, 2.5, 3, 3.5 and 4-year-olds. All participants except for the 4-year-old group were called for a follow-up session that took place 6 months after the first session. Despite families’ willingness to return to the second session, some children could not be evaluated due to a national lockdown during the COVID-19 pandemic (*n* = 14; see Table 2 for sample descriptive statistics).

**Table 2:** Sample descriptive statistics for the first and follow-up session of each age group.

	First session				Follow-up session	
Age group	*n*	Sex	Mean age	*n*	Sex	Mean age
2 years	24	10 males; 14 females	2:0	18	8 males; 10 females	2:6
2.5 years	23	12 males; 11 females	2:6	21	10 males; 11 females	3:0
3 years	32	15 males; 17 females	3:0	22	11 males; 11 females	3:6
3.5 years	32	13 males; 19 females	3:6	29	13 males; 16 females	4:0
4 years	24	16 males; 8 females	4:0	N/A	N/A	N/A

For the mean age the first digit denotes years and the second months. N/A = Not applicable.

### Eye tracker

We used the SensoMotorics Instruments (SMI) RED250 Mobile (Sensomotoric Instruments, 2011) corneal-reflection eye tracker in the current study. Gaze was recorded with iView X Hi-Speed software with a sampling rate of 250 Hz and 0.03° of spatial resolution. A LED LG Flatron E2210PM 22-inch monitor (50–60 Hz) with a native resolution of 1,680 × 1,050 pixel (480 × 300 mm) was used for stimuli display controlled through SMI’s Experiment Center software. Before stimuli presentation, the eye-tracker was calibrated following a five-calibration-point child-friendly procedure in which animated colourful shapes (75 × 75 px) accompanied with melodic sounds were presented in the four corners and centre of the screen. The calibration procedure was repeated in case the child moved or disengaged from the screen, until a successful calibration was achieved. SMI built-in software BeGaze was employed for event detection (saccades and fixations). Peak velocity threshold was set at 40°/s and minimum fixation duration at 50 ms (Conejero and Rueda, 2018). Fixation data was further aggregated with Python 3 custom written code. Scripts generated for data reduction are available from the author.

### Visual sequence learning task

**Figure 1:**
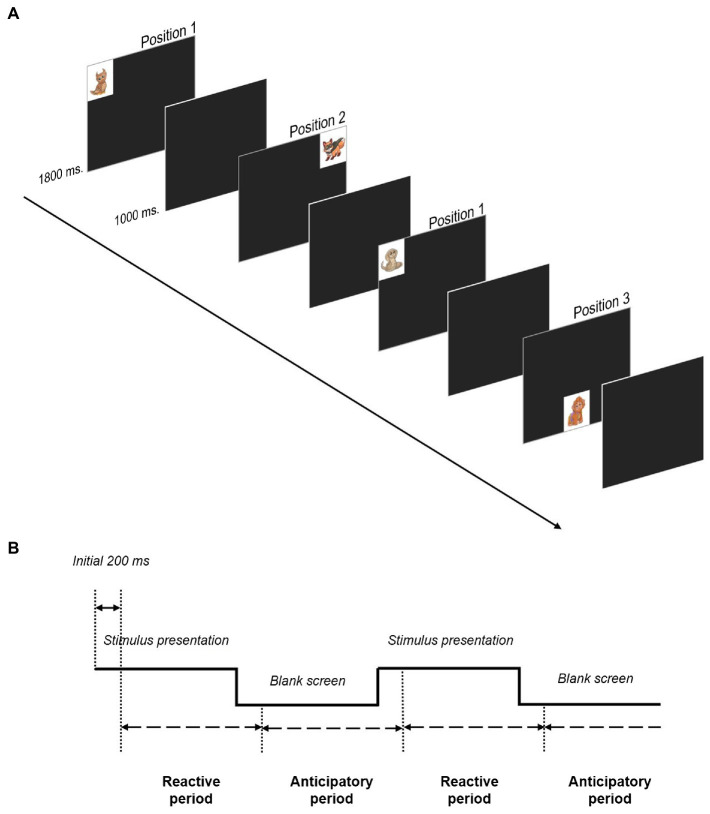
Task procedure of a complete sequence (1-2-1-3) following Clohessy et al. (2001). **(A)** Stimulus are presented in the figure in large size, although a transition through different sizes was employed to create a looming effect. Stimulus presentation (1800 ms) and anticipatory period (1000 ms) durations were fixed in the sequence. Complex (from Position 1-to Position 2 and Position 1-to Position 3) and easy transitions (Position 2-to Position 1, Position 3-to Position 1) can be found in the figure. **(B)** Visualization of the definition of reactive (stimulus presentation) and anticipatory periods (blank screen). Cartoons by GraphicMama-team from Pixabay.

The VSL task consists of the presentation of looming stimuli in a fixed sequence in three locations on the screen: upper right corner (position 1, 13.21° × 4.84° eccentricity to the nearest edge of the full stimulus), upper left corner (position 2, 13.21° × 4.84° eccentricity) and central bottom (position 3, 0° × 4.84° eccentricity) in a specific sequence (1-2-1-3) following Clohessy et al. (2001) (see Figure 1A). Stimuli were presented during 1800 ms and consisted of a dynamic presentation of a picture varying in size (small-medium-small-medium-large stimulus size), to create a looming effect, similarly to Clohessy et al. (2001). The small (4.74°×°4.74° px) and medium (6.65°×°6.65° px) stimuli sizes were presented during 150 ms each to induce the looming effect, while the large size (9.47° × 9.47) remained for 1,200 ms. The stimulus presentation was followed by a blank screen during 1,000 ms that served as the anticipatory period between stimuli. Children were shown a total of 64 trials. The first 12 trials (3 sequences) were used for learning and were not included in the analysis. Thus, a total of 52 experimental trials (13 sequences) were computed for statistical analysis.

Three 20.25°×°14.11 areas of interest (AOI) were defined around each stimulus position in order to compute stimuli fixations, reactive looks and anticipations. The total number of fixations on the stimuli along the duration of the task were coded as stimuli fixations, which provide a measure of active engagement of the participant in the task. In order to identify reactive and anticipatory looks, we defined reactive and anticipatory periods (see Figure 1B). The reactive period started 200 ms after the stimulus onset and ended 200 ms after its disappearance, followed by the anticipatory period which was up to 200 ms after the onset of the following stimulus. Reactive looks are defined as a fixation on the stimulus that occurred during the reactive period, on condition that during the previous anticipatory period the child did not perform a correct anticipatory fixation (in such case the observed fixation on the stimulus would be anticipatory instead of reactive). However, if during the anticipatory period the child does not perform an anticipatory fixation (remains on the same position in which the stimulus had been presented) or performs an incorrect anticipatory fixation, a reactive fixation can occur during the presentation of the upcoming stimulus. Consequently, during the same trial, both an incorrect anticipation and a reactive look could be coded. On the other hand, fixations that occurred during the anticipatory period and were preceded by a stimulus fixation in the previous trial (either reactive or anticipatory) were considered anticipatory looks. As in previous research, anticipatory fixations that were performed in the first 200 ms of the blank screen between stimuli were not considered as such, as they might not reflect real expectations, whereas fixations occurring during the first 200 ms of the stimulus presentation were not considered reactive because the saccade must have been prepared before the stimulus presentation (Canfield and Haith, 1991). Additionally, using the 1-2–1-3 sequence we were able to measure two types of anticipations depending on whether the next stimulus position could be unambiguously predicted from the current position (i.e., position 2 and 3 are always followed by position 1), or the next position is ambiguous and requires monitoring the previous location (i.e., position 1 can be followed by position 2 or 3 depending on the previous position, see Figure 1A). We named these two anticipatory conditions as easy and complex, respectively, which presentation is alternated within the sequence. Participants were not given any instructions or feedback concerning their performance in the task.

We computed the percentage of stimulus fixation over the total number of experimental trials. The proportion of reactive looks and total anticipations were also calculated over the child’s total number of stimulus fixations. Correct and incorrect anticipations reflect an intention to perform anticipatory looks to a location in which something is expected to occur, even if the expectation is not accurate, entailing a voluntary attention shift. In addition, we computed the proportion of correct anticipations based on total anticipations performed, for easy and complex transitions (Rothbart et al., 2003). Children with a percentage of trials with stimuli fixations below 50% (Rothbart et al., 2003) were excluded from further analysis in the first (2-year-olds, *n* = 1; 2.5-year-olds, *n* = 4, 3-years-olds, *n* = 4, 4-year-olds, *n* = 1) and the follow-up session (2.5-year-olds, *n* = 2; 3-year-olds, *n* = 2, 4-year-olds, *n* = 2).

### Parent-reported questionnaires

#### Child temperament

Parents of 2 and 2.5-year-olds children were asked to complete the Spanish Very-Short version of the Early Childhood Behavior Questionnaire (ECBQ; Putnam et al., 2006), while the Children Behavior Questionnaire (CBQ; Rothbart et al., 2003) was filled out by parents of 3-to 4-year-old children. These scales measure 3 temperamental factors: Effortful Control (EC), Surgency (SUR) and Negative Affect (NA). Cronbach’s alpha for EC, SUR and NA for the ECBQ scales were 0.64, 0.62, and 0.42, respectively. Cronbach’s alpha of the NA increased to 0.64 after removing items (10 and 16) with low internal consistency. Cronbach’s alpha of the CBQ were 0.70, 0.76, 0.73, respectively, for EC, SUR and NA.

#### Confusion, hubbub, and order scale

A Spanish adaptation of the CHAOS scale (Matheny et al., 1995) was developed for the purpose of the study. This 15 items scale (α = 0.79) was used to obtain a measure of children’ home chaos. Parents were asked to report their level of agreement with statements that described the organization, environment and family routines at home in a Likert scale ranging from 1 (Completely agree) to 6 (Completely disagree). A final score for home chaos was obtained adding the scores of all the items. Higher scores indicate increased levels of chaos at home.

#### Sociodemographic information

A SES general index was computed averaging the z-scores derived from parents’ education level, professional occupation, and family income-to-needs ratio. Educational level was scored from 1 to 7, following Conejero et al. (2018): 1 (no studies); 2 (elementary school); 3 (secondary school); 4 (high school); 5 (technical degree/university diploma); 6 (university bachelor degree); and 7 (postgraduate studies). Professional occupation was rated following the National Classification of Occupations (CNO-11) of the National Institute of Statistics of Spain (INE) from 0 to 9 as follows: 0 (unemployed); 1 (elemental occupation); 2 (facilities and equipment operators); 3 (manufacturers and construction workers); 4 (qualified professionals in the livestock, agricultural, fishing and forestry sector); 5 (qualified professionals of the restaurant, service and sales industry); 6 (accountant and office workers); 7 (support professionals and technicians); 8 (health, teaching and research professionals;) and 9 (manager). Finally, the income-to-needs ratio was computed dividing the total annual family income by the official poverty threshold provided by the INE considering the number of members in the family unit.

### Procedure

Upon arrival at the laboratory, caregivers were provided with a brief description of the study and asked to complete the informed consent form for participation. The experimental session lasted approximately 1 h and included other lab tasks not reported in the current article. Tasks and questionnaires were administered in a fixed order. Eye tracking tasks were presented first, followed by behavioral tasks. In this sense, children completed first the VSL task while their gaze was recorded with an eye-tracking device. At the end of the session, parents were required to complete temperament, home chaos and SES questionnaires. Due to the close relation between home chaos and SES, the latter environmental factor was also collected to be considered as a control variable. Eye tracking was performed in a semi-dark room, with children seated on a chair at approximately 60 cm from the display monitor. The caregiver was seated nearby behind the child and was instructed to remain silent and to avoid interaction with the child during the administration of the task. The experimenter monitored task administration from an adjacent control room. In order to test changes in attentional abilities and the short-term stability of the measures, the same procedure was repeated in a follow-up session taking place 6 months later and by the same experimenter in the first session. The study obtained ethical approval from the University of Granada Ethics Committee (Ref: 515/CEIH/2018) following the guidelines of the Declaration of Helsinki. Parents received a brief report of the child’s performance and a 10€ voucher for educational toys.

### Analysis plan

Dependent variables were checked for normality and homogeneity of variance. As distributions of stimulus fixations and reactive looks were negatively skewed, a power transformation was applied to improve data distribution. SES was not found to be correlated with neither chaos nor attentional outcomes of the VSL task, so it was not considered as a covariate in subsequent analyses (see Table 3). To investigate the contribution of age, temperament and environmental chaos on attentional performance at Time 1, a series of stepwise regression models were built for each dependent variable. Model building followed the next steps: age was introduced as a continuous variable in the first step of the model, followed by temperamental factors (i.e., effortful control, surgency and negative affectivity) in the second step, and chaos in the third and final step. Considering the change in attentional performance between the first and follow-up session, change scores were computed subtracting performance at Time 1 from Time 2. Similarly, stepwise regression models were built following the same steps previously described, with the only difference being that performance at Time 1 was introduced after age in the second step. Finally, temperamental factors and environmental chaos were introduced in the third and fourth step, respectively.

**Table 3:** Two-tailed partial correlation coefficients, controlling by age, for attentional scores and child’s temperament and environmental factors in the first session.

	1	2	3	4	5	6	7	8	9	10
1. Stimulus fixation	–									
2. Reactive looks	−0.39***	–								
3. Total anticipations	0.33***	−0.84***	–							
4. Easy correct anticipations	−0.14#	−0.42***	0.07	–						
5. Complex correct anticipation	0.16#	−0.09	0.04	−0.50***	–					
6. Effortful control	0.10	0.07	−0.05	−0.01	−0.14	–				
7. Surgency	0.18*	−0.10	0.10	−0.01	−0.06	0.07	–			
8. Negative affect	−0.05	0.03	−0.09	0.03	0.04	0.03	−0.28***	–		
9. Chaos	−0.07	0.01	−0.06	−0.05	0.23**	−0.28***	−0.15#	0.22**	–	
10. SES index	−0.02	0.01	0.03	0.08	−0.13	0.04	−0.04	−0.27**	−0.09	–

***p < 0.001; **p < 0.01; *p < 0.05; #*p* < 0.10.

Associations between temperament and environmental factors with attentional measures were analyzed through two-tailed partial correlations controlling by age for the hypothesized effects. Spearman Rank-Order correlation was applied when any of the dependent variables did not follow the normal distribution.

## Results

### Descriptive statistics

**Figure 2:**
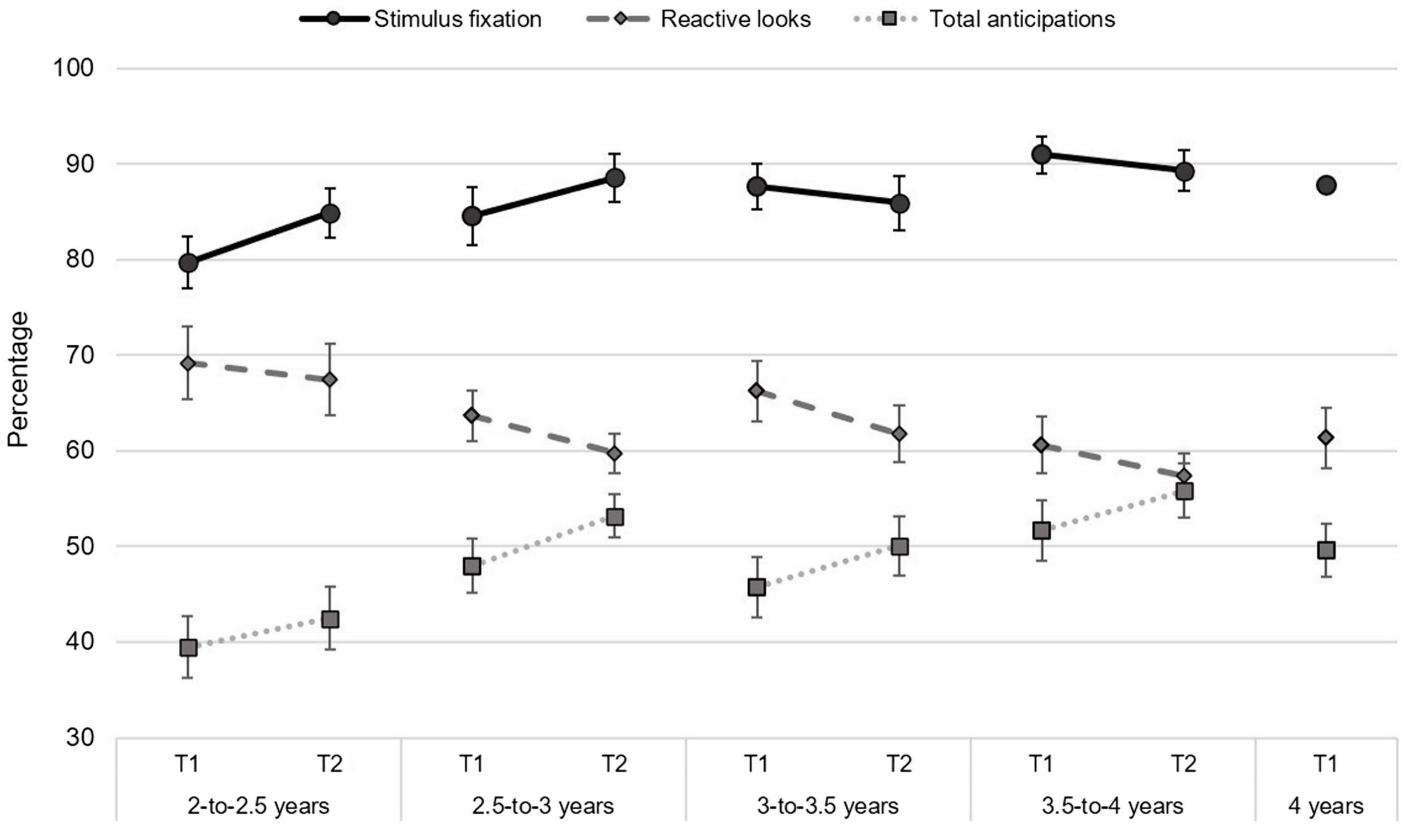
Stimulus fixations, reactive looks and correct anticipations for easy and complex trials. Scores are presented for each age group in the first (T1) and follow-up (T2) sessions. The 4-year-old group was only evaluated in the first session and not followed over time.

**Figure 3:**
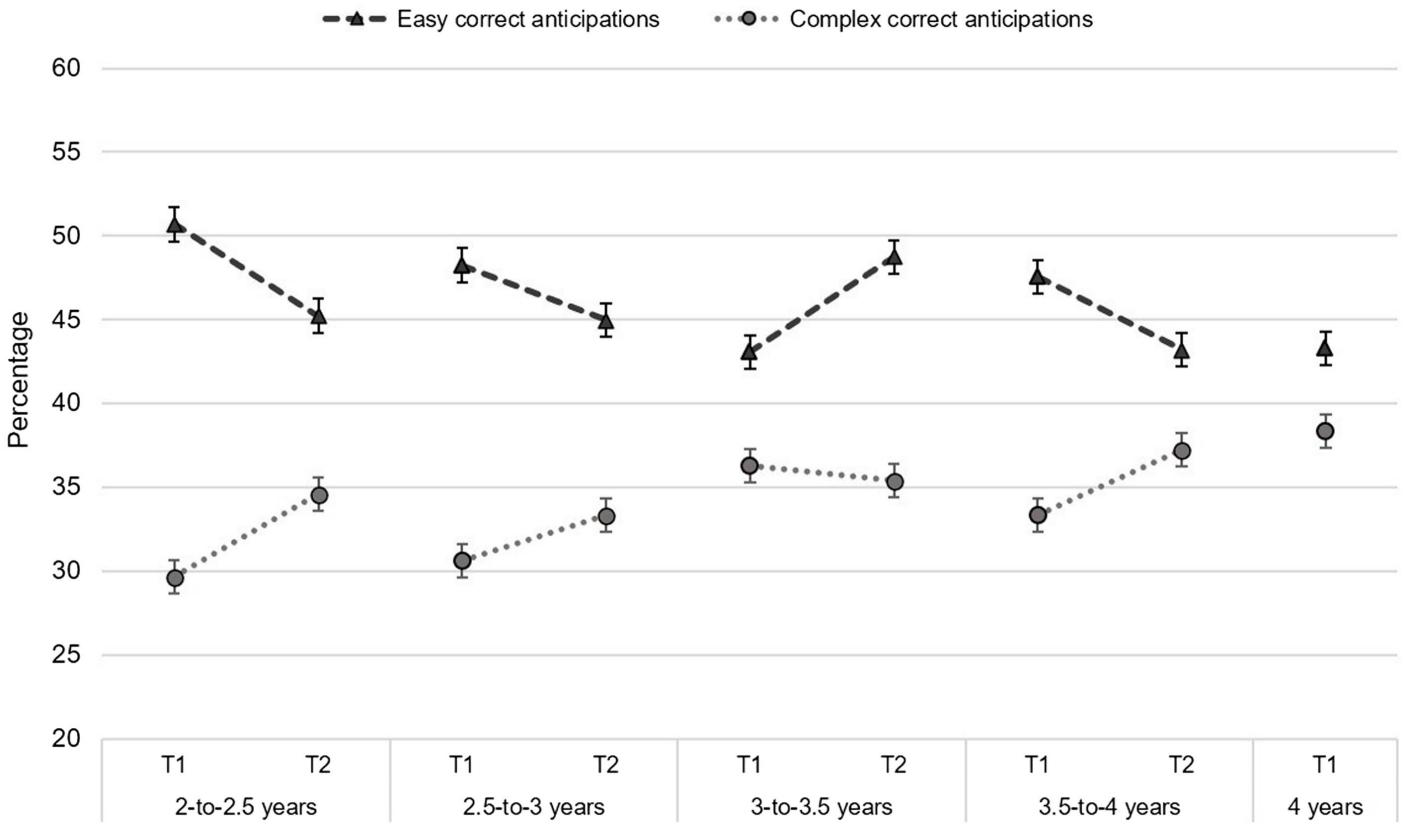
Proportion of easy and complex correct anticipations. Scores are displayed for each age group in the first (T1) and follow-up (T2) session. The 4-year-old group was only evaluated in the first session and not followed over time.

Tables 4, 5 presents descriptive statistics in each age group for questionnaire and VSL measures, respectively. Also, percentages of stimulus fixations, and proportions of reactive looks, total anticipations in function of age group and assessment time are displayed in Figure 2, while correct anticipations for easy and complex transitions are displayed in Figure 3.

**Table 4:** Descriptive statistics for questionnaire measures at Time 1.

Age group	*n* (valid)	Effortful control		Surgency		Negative affect		CHAOS		SES index	
		Mean (SD)	Min (Max)	Mean (SD)	Min (Max)	Mean (SD)	Min (Max)	Mean (SD)	Min (Max)	Mean (SD)	Min (Max)
2 years	24	4.70 (0.73)	3.70 (6.60)	5.67 (0.63)	4.18 (6.91)	3.54 (0.91)	2 (5.70)	43.79 (11.20)	23 (74)	−0.10 (0.94)	−1.35 (1.51)
2.5 years	23	4.82 (0.59)	3.70 (6.10)	5.76 (0.68)	4.58 (6.82)	3.53 (0.74)	2 (5.40)	37.39 (10.37)	22 (64)	−0.27 (0.83)	−1.46 (1.14)
3 years.	32	5.01 (0.63)	3.60 (6.42)	4.57 (0.82)	3 (6)	4.21 (0.87)	2.08 (5.83)	39.93 (9.60)	15 (59)	0.09 (0.92)	−1.51 (1.86)
3.5 years	32	5.49 (0.70)	4 (6.58)	4.60 (0.84)	3.08 (6.50)	4.45 (0.93)	2.33 (6.08)	40.50 (9.56)	23 (55)	−0.09 (0.81)	−1.37 (1.73)
4 years	24	5.32 (0.79)	3.58 (6.58)	4.54 (1.03)	2.42 (6.58)	3.91 (0.80)	2.25 (5)	38.33 (9.44)	19 (56)	0.42 (0.72)	−0.92 (1.90)
Total	135	5.09 (0.75)	3.58 (6.60)	3.98 (0.92)	2 (6.08)	4.97 (0.98)	2.42 (6.91)	40.04 (10.07)	15 (74)	0.009 (0.87)	−1.51 (1.90)

**Table 5:** Descriptive statistics for attentional scores at Time 1 and Time 2.

Age group	Valid *n*		Stimulus fixations (%)		Reactive looks (%)		Total anticipations (%)		Correct anticipations (%)			
									Easy		Complex	
	T1	T2	T1	T2	T1	T2	T1	T2	T1	T2	T1	T2
			Mean (SD)	Mean (SD)	Mean (SD)	Mean (SD)	Mean (SD)	Mean (SD)	Mean (SD)	Mean (SD)	Mean (SD)	Mean (SD)
2 years	23	16	79.68 (10.76)	84.85 (10.48)	69.18 (14.97)	67.39 (14.98)	39.43 (12.91)	42.47 (13.18)	50.68 (17.64)	45.22 (19.49)	29.62 (15.58)	34.57 (11.53)
2.5 years	23	17	84.50 (12.43)	88.57 (10.36)	63.63 (10.70)	59.72 (8.51)	47.98 (11.95)	53.17 (9.17)	48.25 (10.87)	44.94 (12.99)	30.61 (9.43)	33.32 (7.78)
3 years.	28	19	87.65 (10.32)	85.93 (12.31)	66.23 (13.58)	61.75 (13.01)	45.71 (13.87)	50.09 (13.48)	43.08 (13.09)	48.74 (15.85)	36.30 (8.70)	35.38 (11.62)
3.5 years	28	21	90.93 (8.90)	89.28 (9.57)	60.61 (13.39)	57.36 (10.99)	51.68 (14.50)	55.80 (13.03)	47.57 (14.49)	43.18 (10.71)	33.36 (8.42)	37.21 (6.44)
4 years	23	N/A	87.71 (11.86)	N/A	61.33 (15.26)	N/A	49.58 (13.16)	N/A	43.31 (12.33)	N/A	38.32 (6.20)	N/A
Total	125	73	86.12 (11.15)	87.27 (10.65)	64.65 (13.35)	61.25 (12.34)	46.58 (13.88)	50.78 (13.10)	47.24 (14.14)	45.48 (14.69)	32.66 (10.76)	35.25 (9.41)

T1 = Time 1; T2 = Time 2; N/A = Not applicable. Stimulus fixations are computed over the number of experimental trials. Reactive looks and total anticipations are computed as a proportion of stimulus fixations. Correct easy and complex anticipations are calculated as a proportion of total anticipations.

### Stimulus fixations

Regarding stimulus fixations at Time 1, the model for the first step including age was found to be statistically marginal (*R*^2^= 0.03, *F*(1, 123) = 3.93, *p* = 0.05), with age being a marginal predictor (*β* = 0.18, *p* = 0.05, 95% CI [0.06, 82.26]). Adding temperamental factors in the second step (∆*R*^2^= 0.03, ∆*F*(3, 120) = 1.35, *p* = 0.26) and chaos in the final step (∆*R*^2^< 0.01, ∆*F*(1, 119) = 0.36, *p* = 0.55) did not significantly increase the variance explained by the model. The final model explained 6% of the total variance for stimulus fixations at Time 1 (Table 6). Considering the change in stimulus fixations between Time 1 and Time 2, the model in the first step with age as a predictor was found statistically marginal (*R*^2^= 0.04, *F*(1, 71) = 3.19, *p* = 0.08) with age being a statistically marginal predictor (*β* = −0.21, *p* = 0.08, 95% CI [−149.05, 8.16]). Introducing performance at Time 1 returned a statistically significant change in the model (∆*R*^2^= 0.37, ∆*F*(1, 70) = 43.65, *p* < 0.01). Only previous performance was found as a statistically significant predictor (*β* = −0.65, *p* < 0.01, 95% CI [−1.03, −0.55]). Neither model change for the third step adding temperamental factors (∆*R*^2^= 0.04, ∆*F*(3, 67) = 1.59, *p* = 0.20) nor in the fourth step including chaos (∆*R*^2^< 0.01, ∆*F*(1, 66) = 0.85, *p* = 0.36) were found statistically significant. The final model explained 46% of the total variance for the change in stimulus fixations (Table 7).

**Table 6:** Regression coefficients for stimulus fixations, reactive looks and total anticipations at Time 1.

	Stimulus fixations			Reactive			Total anticipations		
	Step 1	Step 2	Step 3	Step 1	Step 2	Step 3	Step 1	Step 2	Step 3
	*β*			*β*			*β*		
1. Child variable									
Age	0.18#	0.26*	0.25*	−0.19*	−0.28*	−0.28*	0.18*	0.27*	0.26*
2. Temperament									
Effortful Control	–	0.04*	0.02	–	0.08	0.08	–	−0.06	−0.07
Surgency	–	0.20#	0.20#	–	−0.12	−0.12	–	0.10	0.10
Negative Affectivity	–	0.02	0.03	–	< 0.01	< 0.01	–	−0.06	−0.05
3. Environment									
CHAOS	–	–	−0.06	–	–	< 0.01	–	–	−0.06
∆R^2^	0.03	0.03	< 0.01	0.03	0.02	< 0.01	0.03	0.02	< 0.01
∆F	3.93#	1.65	0.36	4.52*	0.70	< 0.01	4.40*	0.73	0.37

***p < 0.001; **p < 0.01; *p < 0.05; #*p* < 0.10.

**Table 7:** Regression coefficients for change between Time 1 and Time 2 for stimulus fixations, reactive looks, and total anticipations.

	Stimulus fixations				Reactive looks				Total anticipations			
	Step 1	Step 2	Step 3	Step 4	Step 1	Step 2	Step 3	Step 4	Step 1	Step 2	Step 3	Step 4
	*β*				*β*				*β*			
1. Child variable												
Age	−0.21#	0.03	−0.07	−0.08	−0.04	−0.17#	−0.26*	−0.24#	0.02	0.19#	0.30*	0.29*
2. Previous performance												
T1 performance	–	−0.65***	−0.65***	−0.65***	–	−0.63***	−0.64***	−0.63***	–	−0.61***	−0.61***	−0.62***
3. Temperament												
Effortful Control	–	–	0.22*	0.19#	–	–	0.04	0.11	–	–	0.03	< 0.01
Surgency	–	–	−0.07	−0.07	–	–	−0.10	−0.08	–	–	0.22#	0.21#
Negative Affectivity	–	–	−0.06	−0.03	–	–	0.06	< 0.01	–	–	−0.01	0.02
4. Environment												
CHAOS	–	–	–	−0.09	–	–	–	0.20#	–	–	–	−0.11
∆R^2^	0.03	0.39	0.41	0.41	< 0.01	0.38	0.01	0.03	< 0.01	0.34	0.04	0.01
∆F	3.19#	43.65***	1.59	0.85	0.09	42.86***	0.52	3.70#	0.03	36.78***	1.34	1.07

***p < 0.001; **p < 0.01; *p < 0.05; #*p* < 0.10.

T1 = Time 1.

### Reactive looks

For reactive looks at Time 1, the model for the first step including age was found statistically significant (*R*^2^= 0.03, *F*(1, 123) = 4.52, *p* = 0.04), with age being predictive of reactive looks (*β* = −0.19, *p* = 0.04, 95% CI [−75.60, −2.70]). None of the subsequent steps led to a significant change in the model (all *ps* > 0.55). A total 5% of the variance for reactive looks at Time 1 was explained by the full model (Table 6). For the change in reactive looks between sessions, the first step in the model including only age as predictor was not found to be statistically significant (*R*^2^< 0.01, *F*(1, 71) = 0.09, *p* = 0.76). However, including performance at Time 1 led to a statistically significant change in the variance explained by the model (∆*R*^2^= 0.38, ∆*F*(1, 70) = 42.86, *p* < 0.01). Age was found to be a statistically marginal predictor (*β* = −0.16, *p* = 0.09, 95% CI [−94.14, 6.67]) of change in reactive looks, while performance at Time 1 was found statistically significant (*β* = −0.63, *p* < 0.01, 95% CI [−0.83, −0.44]). Model change for the third step including temperamental factors was not found to be statistically significant (∆*R*^2^= 0.01, ∆*F*(3, 67) = 0.52, *p* = 0.67), while for the final step adding chaos was found statistically marginal (∆*R*^2^= 0.03, ∆*F*(1, 66) = 3.70, *p* = 0.06). In this case, previous performance at Time 1 was found to be a statistically significant predictor (*β* = −0.63, *p* < 0.01, 95% CI [−0.83, −0.44]), while age (*β* = −0.24, *p* = 0.06, 95% CI [−127.77, 2.87]) and chaos (*β* = 0.19, *p* = 0.06, 95% CI [−1.26, 68.35]) were found to be statistically marginal predictors. This final model explained 43% of the variance for change in reactive looks (Table 7).

### Total (correct and incorrect) anticipations

Concerning total anticipations, the model for the first step including age was found to be statistically significant (*R*^2^= 0.03, *F*(1, 123) = 4.40, *p* = 0.04), with age being predictive of total anticipations (*β* = 0.19, *p* = 0.04, 95% CI [0.02, 0.60]). However, none of the following steps increased the variance explained by the model (all *ps* > 0.53). The full model explained 5% of the total variance for total anticipations (Table 6). The first step for the model predicting the change in total anticipations including only age was not found to be statistically significant (*R*^2^< 0.01, *F*(1, 71) = 0.03, *p* = 0.87). Introducing previous performance at Time 1 led to a statistically significant change in the model (∆*R*^2^= 0.34, ∆*F*(1, 70) = 36.78, *p* < 0.01). In this step, age was found to be a marginal predictor of change in total anticipations (*β* = 0.19, *p* = 0.06, 95% CI [−0.06, 0.82]), while previous performance at Time 1 was a significant predictive variable (*β* = −0.61, *p* < 0.01, 95% CI [−0.82, −0.41]). Subsequent changes in the model were not found to be statistically significant (all *ps* > 0.27). The final model explained 39% of the total variance of change in total anticipations (Table 7).

### Correct anticipations

Models were built for both easy and complex anticipations. For easy correct anticipations, none of the steps were found to be statistically significant (all *ps* > 0.50), with the full model explaining only 0.08% of the total variance for easy correct anticipations. Concerning complex correct anticipations, the model in the first step with only age as a predictor was found to be statistically significant (*R*^2^= 0.05, *F*(1, 123) = 6.94, *p* = 0.01). Age was found to be predictive of complex correct anticipations (*β* = 0.23, *p* = 0.01, 95% CI [0.07, 0.49]). Including temperamental factors in the second step did not lead to a significant change in the model (∆*R*^2^= 0.02, ∆*F*(3, 120) = 0.96, *p* = 0.41), but adding chaos in the third step did (∆*R*^2^= 0.03, ∆*F*(1, 119) = 4.63, *p* = 0.03). For this full model, both age (*β* = 0.27, *p* = 0.01, 95% CI [0.07, 0.58]) and chaos (*β* = 0.20, *p* = 0.03, 95% CI [0.02, 0.37]) were found to be statistically significant predictors of complex correct anticipations. The final model explained 11% of the total variance for complex correct anticipations (Table 8).

**Table 8:** Regression coefficients for correct anticipations at Time 1.

	Correct easy anticipations			Correct complex anticipations		
	Step 1	Step 2	Step 3	Step 1	Step 2	Step 3
	*β*			*β*		
1. Child variable						
Age	−0.05	−0.05	−0.06	0.23*	0.25*	0.27*
2. Temperament						
Effortful Control	–	< 0.01	−0.03	–	−0.14	−0.09
Surgency	–	< 0.01	< 0.01	–	−0.04	−0.03
Negative Affectivity	–	0.03	0.05	–	0.03	−0.01
3. Environment						
CHAOS	–	–	−0.07	–	–	0.20*
∆R^2^	< 0.01	< 0.01	< 0.01	0.05	0.02	0.03
∆F	0.35	0.04	< 0.01	6.94*	0.96	4.63*

***p < 0.001; **p < 0.01; *p < 0.05; #*p* < 0.10.

Likewise, models were built for the change in easy and complex correct anticipations. For easy correct anticipations, the model in the first step including age was not found to be statistically significant (*R*^2^< 0.01, *F*(1, 71) = 0.23, *p* = 0.63). Introducing previous performance at Time 1 in the model led to a statistically significant change (∆*R*^2^= 0.47, ∆*F*(1, 70) = 62.02, *p* < 0.01). Only previous performance at Time 1 was found to be a statistically significant predictor (*β* = −0.68, *p* < 0.01, 95% CI [−1.23, −0.73]). Including temperament in the third step of the model led to a statistically marginal change (∆*R*^2^= 0.05, ∆*F*(3, 67) = 2.35, *p* = 0.08), with previous performance in Time 1 being found to be a statistically significant predictor (*β* = −0.65, *p* < 0.01, 95% CI [−1.18, −0.68]), along with a statistically marginal predictive effect of effortful control (*β* = −0.19, *p* = 0.05, 95% CI [−10.69, −0.05]). Adding chaos in the final step of the model did not significantly increase the variance explained (∆*R*^2^= 0.02, ∆*F*(1, 66) = 2.36, *p* = 0.13). Concerning the change in complex correct anticipations, the first step with age as a predictor was also not found statistically significant (*R*^2^< 0.01, *F*(1, 71) = 0.27, *p* = 0.61), but the change in the model for the second step including previous performance at Time 1 was (∆*R*^2^= 0.57, ∆*F*(1, 70) = 92.71, *p* < 0.01). Only previous performance at Time 1 was found to be a significant predictor (*β* = −0.77, *p* < 0.01, 95% CI [−1.23, −0.81]). Subsequent steps did not change the model significantly (all *ps* > 0.38). Both full models for the change in easy and complex correct anticipations explained 54% of the total variance in each case (Table 9).

**Table 9:** Regression coefficients for change between Time 1 and Time 2 for correct anticipations.

	Correct easy anticipations				Correct complex anticipations			
	Step 1	Step 2	Step 3	Step 4	Step 1	Step 2	Step 3	Step 4
	*β*				*β*			
1. Child variable								
Age	0.06	−0.01	0.01	<0.01	−0.06	0.08	0.07	0.08
2. Previous performance								
T1 performance	–	−0.69***	−0.65***	−0.65***	–	−0.77***	−0.76***	−0.77***
3. Temperament								
Effortful Control	–	–	−0.20*	−0.24*	–	–	0.05	0.07
Surgency	–	–	−0.13	−0.14	–	–	0.04	0.05
Negative Affectivity	–	–	−0.04	< 0.01	–	–	0.04	0.01
4. Environment								
CHAOS	–	–	–	−0.14	–	–	–	0.08
∆R^2^	< 0.01	0.47	0.05	0.02	< 0.01	0.57	< 0.01	< 0.01
∆F	0.23	62.02***	2.35	2.36	0.27	92.71***	0.24	0.77

***p < 0.001; **p < 0.01; *p < 0.05; #*p* < 0.10.

T1 = Time 1.

### Correlation analyses

Correlation analyses between temperament and attention revealed only a significant positive relation between stimulus fixations and temperamental surgency (*r* = 0.18, *p* = 04, 95% CI [0.01, 0.35]). Concerning environmental variables, a statistically significant positive correlation was found between correct complex anticipations and chaos (*r* = 0.23, *p* < 0.01, 95% CI [0.06, 0.39]). No other statistically significant correlations of attention with temperamental or environmental factors were found (see Table 3). Intercorrelations of task measures across sessions are reported in Table 10.

**Table 10:** Two-tailed partial correlation coefficients, controlling by age, for VLS scores in the first and follow-up sessions.

	1. T2	2. T2	3. T2	4. T2	5. T2
1. T1: Stimulus fixation	0.22#	−0.17	0.18	0.18	−0.05
2. T1: Reactive looks	−0.04	0.36***	−0.34**	−0.32**	0.06
3. T1: Total anticipations	0.10	−0.44***	0.41***	0.26*	−0.04
4. T1: Easy correct anticipations	−0.13	−0.06	−0.07	0.02	0.09
5. T1: Complex correct anticipation	−0.09	−0.12	0.20#	0.16	−0.02

***p < 0.001; **p < 0.01; *p < 0.05; #*p* < 0.10.

T1 = Time 1; T2 = Time 2.

## Discussion

The goal of the present research was to study age differences in increasingly complex forms of visual attention control, from exogenous to endogenous orienting and context monitoring. The VSL task provides measures that allow for the analysis of these changes. Anticipatory gaze is conceptualized as a measure of endogenous orienting of attention that is based on the development of an expectation of where something is expected to occur. When these expectations require context monitoring, endogenous orienting has been shown to rely on executive control mechanisms (Curran and Keele, 1993). Previous results with the VSL task contributed to state the hypothesis that a transition from basic forms of endogenous orienting towards context monitoring, involving executive attention, emerge during toddlerhood (Rothbart et al., 2003). However, to our knowledge studies on changes of these specific forms of visual attention control between toddlerhood and the preschool period had not yet been addressed. In the current study, age differences were examined with a cross-sequential design mixing within-and between-subjects effects. Different cohorts of children between 2 and 4 years of age were evaluated in two sessions placed 6 months apart.

### Development of visual attention control

The percentage of stimulus fixations can be considered indicative of children’s sustained attention, as it reflects their active engagement in the task over time. Contrary to our hypothesis, we did not find significant age-related changes in sustained attention. Although an age-related tendency for stimulus fixations to increase with age in the first session was found, the effect was not statistically significant. Increases with age in this attentional ability has been previously reported in toddlers (Ruff et al., 1998) and preschoolers (Graziano et al., 2011). These studies suggest that during these developmental stages, children are in the process of increasing their ability to remain task-engaged for a sustained period of time. The development of this sustained attention shows further enhancements along childhood for tasks of progressively longer durations (Betts et al., 2006). We only observed marginally significant age-associated changes in stimulus fixations between sessions. This effect of age was lost once performance in the first session was introduced in the model, accounting for most of the variance. It should be noted that stimulus fixations are already high the first-time children are exposed to the task, with 2-year-olds displaying 80% and 4-year-olds 88%. Perhaps differences in stimulus variability and presentation rate could have facilitated children to remain engaged, in comparison to other experimental procedures. However, children higher tendency to remain engaged in the task already in the first session, left less margin for change in a 6 months period.

Exogenous orienting was examined through reactive looks towards displayed stimuli, as a form of bottom-up orienting of attention. A significant decrease in reactive looks with age was found in the first session. Exogenous attention provides a generic mechanism to acquire basic knowledge of a novel context, which is in place from the first months of life. As children gain experience with the sequence and become better at anticipating the upcoming location of the target, the percentage of reactive looks is reduced. In fact, there is a negative correlation between the percentage of reactive and anticipatory looks both within and across sessions (see Tables 3, 10). Indeed, this might explain the reduction with age for reactive looks observed in the first session of our study, as a greater ability to engage anticipatory attention (total anticipations) is found with age in the same session. Although no age differences in reactive looks were anticipated, given that this form of orientation develops very early on (Colombo, 2001), it is expected that an increased capacity to anticipate gaze would result in reduced number of reactive looks as children engage in a more proactive orienting of attention. Employing a similar version of the VSL, Sheese et al. (2008) reported a higher percentage of reactive looks (79% on average) for 6-to 7-month-old infants, in comparison to the youngest (69%) and oldest (61%) cohorts of our sample. This likely reflects infants’ reduced capacity for endogenous control, engaging in a more exogenous orienting of attention. Considering the change between sessions, age was only found to marginally predict a reduction in reactive looks once accounting for children’s initial performance. One possible explanation is that the temporal gap between sessions could be too short for attentional changes to emerge at these ages. This idea is also supported by the marginal effect of age in the change of total anticipations between sessions. As both measures are negatively correlated, it suggests a trade-off between exogenous (reactive looks) and endogenous orienting (total anticipations). As stated before, perhaps a six-month window could be too narrow for differences in endogenous control to emerge, undermining changes in exogenous attention.

Endogenous orienting was measured with anticipatory looks, both total and correct. Total anticipations provide information about children’s voluntary effort to anticipate, independently of the accuracy of their formed expectations. In line with our hypothesis, an increase in total anticipations with age was found in the first session. The oldest children of the sample showed a greater percentage of anticipatory looks compared to the younger groups, which reflects their increased capacity for endogenous control of orienting. When exposed to the sequence of stimuli for the first time, children might adopt an exploratory strategy in order to learn the underlying contingencies of the sequence. Children’s exploratory behaviour is thought to rely on endogenous orienting at the beginning of the task, in an effort to make sense of the environment they are exposed to Clohessy et al. (2001). As more sequences are repeated, children’s attentional orienting is gradually internalized, moving towards a more proactive approach, increasing attempts to anticipate upcoming events (Chatham et al., 2009). These voluntary attempts would allow them to gather more precise information in an effort to engage in an active monitoring of the sequence. Once contextual information is acquired, it is used to deploy attention to spatially relevant aspects of the current visual context (Chun and Jiang, 1998) in order to correctly anticipate.

Correct anticipations also provide a measure of endogenous orienting, tapping into the learning of the sequence. Therefore, it is a measure of the active engagement of the child in the monitoring of the context to maximize the accuracy of the formed expectations. The sequence used in this study includes two types of transitions, those that are easy to track (i.e., unambiguous transitions: from location 2 or 3 the next stimulus always appears in location 1), and those that are more complex to track because they require monitoring the previous locations (i.e., ambiguous transitions: from location 1 the next stimulus location depends on what the previous one was). No age-related increases in correct anticipations were found for easy transitions. Therefore, children in the studied age range are equally able to predict the occurrence of a forthcoming event when learning relies on unambiguous contextual information. This result is not surprising, as infants and toddlers have been previously found to perform a similar number of anticipations in easy transitions as adults (Clohessy et al., 2001). At older ages, no differences have been found between 2 and 3-year-olds for these transitions or even between two blocks of the task (Rothbart et al., 2003). This indicates that toddlers are already able to learn this type of transitions early in the task, and that this ability remains relatively stable until, at least, the third year. The current results contribute to extend the stability for anticipations in context free settings up to 4 years of age. Additionally, age was not found to predict the change for easy correct anticipations between sessions. This emphasizes that those children with an initial high performance during the first session would increase less in the follow-up. These are straightforward findings, as children from the different studied cohorts were found to be equally able to anticipate and learn from easy transitions already during the first exposure to the task. Consequently, the change in the follow-up session could be expected not to change significantly in a six-month window.

As hypothesized, we observed a significant increase in complex correct anticipations with age. Similar results were previously observed by Rothbart et al. (2003) between cohorts of 2 and 3 years of age, observing an increase in complex correct anticipations. The current results replicate this finding, but also extend the period of development of context-dependent learning up to 4 years. We found a progressive increase with age in correct anticipations for complex transitions (from 30% at 2 years of age to 38% at 4 years of age in T1; see Table 5 and Figure 2). This suggests that 2-year-olds are less able to engage executive control mechanisms to monitor the context and control the orientation of attention accordingly, with this ability significantly progressing in the following years. Previous studies have also shown an important development of executive attention skills between 2 and 4 years of age, although most of them examine the development of action-regulation mechanisms, such as inhibitory control and cognitive flexibility (Gerardi-Caulton, 2000; Jones et al., 2003; Blakey et al., 2016). On the other hand, age was not found to predict the change in complex correct anticipations but only performance during the first session. To our knowledge, no prior study has examined the differences in context-dependent visual sequences in relation to control over orienting of attention between toddlerhood and preschool ages. This ability is strongly dependent on the development of fronto-parietal regions involved in top-down control of attention (Corbetta et al., 2008), which show a protracted developmental trajectory extending beyond childhood (Power et al., 2010). The proper adjustment of attention orientation to anticipate in complex transitions requires an important dose of sustained top-down attention as well as working memory in order to hold previous locations in mind in order to be able to predict the upcoming one. Using a different sequential task than the one used in this study, prior research has found that it is not until the end of the preschool period when children display a better capacity to monitor sequences of actions (Freier et al., 2017). The current findings also support this view, although applied to the monitoring of visual sequences, highlighting an improvement in the engagement of executive attention control from 2-to 4 years of age.

### Individual differences in visual attention

A secondary goal of the study was to examine the individual stability of visual attention and its association with child temperament and home environment, as potential factors with an impact on early attention. Concerning the stability of the measures, our results revealed positive correlations for reactive looks and total anticipations taken 6 months apart. As the ability to voluntarily control attention and recruit executive control mechanisms is suggested to be under development at these ages, processes more stablished as exogenous and more basic forms of endogenous control of orienting could show a higher stability. Regarding intercorrelations in the first session between attentional measures, we found a higher exogenous orienting (i.e., reactive looks) to be negatively associated with endogenous control of visual attention (i.e., less stimulus fixations, total anticipations and easy correct anticipations). Children with a more reactive approach seem more likely to disengage from the task and less prone to engage in a proactive anticipation of stimuli. This pattern is also observed between sessions, as reactive fixations in the first session were negatively correlated with total and easy correct anticipations in the follow-up. In this sense, a higher reliance on exogenous orienting may harm the learning of the sequence not only concurrently, but also in a period of 6 months. On the other hand, a higher sustained attention (i.e., higher percentage of stimulus fixations along the task) was positively correlated with a higher endogenous control (i.e., total anticipations), showing only a tendency to also be positively associated with the engagement of monitoring processes (i.e., complex correct anticipations). In general, these results highlight a trade-off between exogenous and endogenous attention, allowing for a clear dissociation between these processes. Furthermore, some indices of endogenous orienting were intercorrelated, suggesting that endogenous and executive control of attention, both necessary for context monitoring, support each other as mechanisms underlying the development of voluntary control.

Results show temperament to not contribute significantly to increase the variance already explained by age on measures of visual attention. Surprisingly, individual differences seem to have a limited role in capturing variability of children’s attentional abilities in this paradigm in the age range of study. Moreover, the hypothesized association between attention control and effortful control was not found. This suggests that the VSL task could be more permeable to the effects of contextual information in attention control rather than individual differences in self-regulatory abilities. Nevertheless, our data also yielded two general associations. On one hand, we found a positive correlation between surgency and sustained attention. Dimensions of surgency, such as extraversion and positive approach are known to be related to a higher proneness to respond to external stimulation. This could derive in an attentive style that may predispose children to engage in a more exploratory behavior and active engagement with the task. Infants with high surgency scores have also found to exhibit greater cognitive control, displaying shorter fixation durations which may be linked to a faster information encoding (Papageorgiou et al., 2014, 2015). At older ages, this temperamental trait has also been positively associated with sustained attention in preschoolers, presumably due to a positive task involvement (Rothbart and Posner, 2006).

Contrary to our hypothesis, we found home chaos to positively contribute to predict correct anticipations for complex transitions after accounting for age and temperament. This is an interesting result, as it may imply that children exposed to less predictable environments at home would show certain advantage when learning contingencies from the environment. Children exposed to more unorganized and unpredictable households could had increased their vigilance towards external events, engaging in a greater effort to try to create expectations on a daily basis. When exposed to more predictable conditions, this could constitute an advantage. In this regard, the temporal and predictable structure of the sequences in the task could ease the engagement of top-down control in these children, allowing them to orient attention towards information-rich aspects (Wass, 2022). Hence, they could outperform children with a lower exposure to unpredictable environments when required to learn context-dependent information. Nevertheless, this an open interpretation that needs replication in future research. The lack of correlation between chaos and SES supports the idea that chaos can be distributed across different SES backgrounds, accounting as a more proximal factor to cognitive changes during early development (Valiente et al., 2007; Marsh et al., 2020). In this sense, previous research with preschoolers and school-age children have found chaos to predict independent effects on cognition than those of SES (Petrill et al., 2004; Hart et al., 2007). The current findings contribute to support this notion, as well to the understanding of the differential impact of home chaos over attentional abilities during early development.

Finally, we found a consistent relation between child’s temperament and chaos at home. We observed an increased negative affectivity and lower effortful control to be associated with children raised in families scoring high in home chaos. This is congruent with previous data showing a negative association between SES and toddlers’ negative affectivity (Conejero and Rueda, 2018). The negative associations of chaos with effortful control but positive with complex correct anticipation was surprising. This could imply that chaos could have a negative impact on the child’s self-regulatory abilities and general attention control measured through effortful control. However, it could offer certain advantages when children are required to control attention more efficiently to learn from the context. Future studies should test this interpretation, also exploring how children raised at different levels of chaos at home are able to dismiss random noise within the sequence, especially for context-dependent transitions. Perhaps children exposed to higher levels of chaos will be less affected by this noise, as it could resemble the reduced number of contingent events that characterize disorganized households. Similarly, it would be important to consider children’s awareness of the sequence and its association with correct anticipations in easy and complex transitions. As the latter would rely to a greater extent on an explicit knowledge of the sequence, it would be more dependent on executive attention resources.

The current study is not free of limitations. Although over 150 children took part in the study, five age groups were considered, turning out to be a limited sample size of approximately 20 children per cohort. Increasing the statistical power may help to clarify some tendencies in attentional measures found in the data. Concerning the anticipatory period, the chosen interval was fixed at 1000 ms, while previous studies have used times around 750 ms (Clohessy et al., 2001; Rothbart et al., 2003). This increased anticipatory period could have facilitated the processing of information and planning of anticipatory eye movements. It would be desirable for future studies to compare different anticipatory periods. This would help to disentangle if the time needed to plan and execute anticipatory looks could have an effect on age differences, especially when considering different cohorts.

The key strength of the current study lies in the combination of mixed within-and between-subjects effects in a cross-sequential design, as well as the consideration of a wide range of ages. This approach facilitates to compare several age groups and to follow each cohort over time to test attentional changes and consider the stability of the attentional measures. Furthermore, employing eye-tracking technology was a technical improvement to the study of visual attention control with the VSL, compared to offline recordings employed in previous studies. This contributes to gain in temporal and spatial precision in the measures of visual attention derived from the task. Finally, although we found a contribution of temperament and home chaos to visual attention control, the variance explained by these factors remain relatively low. It is likely that other key factors could add to explain additional variance at these ages. Individual variations in dopamine genes, such as the dopamine transporter type 1 (DAT1), have been reported to be associated with the development of attention control during childhood. Specifically, those children with the long allele of the DAT1 show greater attention control (Rueda et al., 2005). Additionally, considering other elements of children’s environment, especially those found to be closely related to cognitive functioning in the first years, such as children’s nutrition, quality of sleep or physiological stress (Roosa et al., 2005), could add to increase the understanding of the effects of the early environment on visual attention control.

## Data availability statement

The raw data supporting the conclusions of this article will be made available by the authors, without undue reservation.

## Ethics statement

The studies involving human participants were reviewed and approved by University of Granada Ethics Committee. Written informed consent to participate in this study was provided by the participants’ legal guardian/next of kin.

## Author contributions

SM and MR conceived and designed the study. SM, ÁC, and MR wrote the manuscript. SM coordinated and acquired the data, and conducted data analyses. FS contributed to study design and reviewed the manuscript. MF helped with data acquisition. All authors contributed to the article and approved the submitted version.

## Funding

This work was supported by a grant from the Spanish Ministry of Science and Innovation (MINCINN; Ref: PSI2017-82670-P) awarded to MR and a FPI fellowship from the Spanish Ministry of Science and Innovation (MINCINN; Ref: PRE2018-083592) awarded to MS.

## Conflict of interest

The authors declare that the research was conducted in the absence of any commercial or financial relationships that could be construed as a potential conflict of interest.

## Publisher’s note

All claims expressed in this article are solely those of the authors and do not necessarily represent those of their affiliated organizations, or those of the publisher, the editors and the reviewers. Any product that may be evaluated in this article, or claim that may be made by its manufacturer, is not guaranteed or endorsed by the publisher.
